# Single Dose of N-Acetylcysteine in Local Anesthesia Increases Expression of HIF1α, MAPK1, TGFβ1 and Growth Factors in Rat Wound Healing

**DOI:** 10.3390/ijms22168659

**Published:** 2021-08-12

**Authors:** Wiktor Paskal, Michał Kopka, Albert Stachura, Adriana M. Paskal, Piotr Pietruski, Kacper Pełka, Alan E. Woessner, Kyle P. Quinn, Ryszard Galus, Jarosław Wejman, Paweł Włodarski

**Affiliations:** 1 Department of Methodology, Medical University of Warsaw, 02-091 Warsaw, Poland; m_kopka@wp.pl (M.K.); albert.stachura@wum.edu.pl (A.S.); adriana.paskal@gmail.com (A.M.P.); kacper.pelka@wum.edu.pl (K.P.); pawel.wlodarski@wum.edu.pl (P.W.); 2 Doctoral School, Medical University of Warsaw, 02-091 Warsaw, Poland; 3 Centre of Postgraduate Medical Education, Department of Replantation and Reconstructive Surgery, Gruca Teaching Hospital, 05-400 Otwock, Poland; pietruski.piotr@gmail.com; 4 Department of Biomedical Engineering, University of Arkansas, Fayetteville, AR 72701, USA; aewoessn@email.uark.edu (A.E.W.); kpquinn@uark.edu (K.P.Q.); 5 Department of Histology and Embryology, Medical University of Warsaw, 02-091 Warsaw, Poland; ryszard.galus@wum.edu.pl; 6 Department of Pathology, Centre of Postgraduate Medical Education, 00-416 Warsaw, Poland; jarwej@poczta.fm

**Keywords:** wound healing, skin, regeneration, N-Acetylcysteine, therapy, growth factor, rat, qPCR, local anesthesia additive, incision

## Abstract

In this study, we aimed to investigate the influence of N-acetylcysteine (NAC) on the gene expression profile, neoangiogenesis, neutrophils and macrophages in a rat model of incisional wounds. Before creating wounds on the backs of 24 Sprague–Dawley rats, intradermal injections were made. Lidocaine–epinephrin solutions were supplemented with 0.015%, 0.03% or 0.045% solutions of NAC, or nothing (control group). Scars were harvested on the 3rd, 7th, 14th and 60th day post-surgery. We performed immunohistochemical staining in order to visualize macrophages (anti-CD68), neutrophils (anti-MPO) and newly formed blood vessels (anti-CD31). Additionally, RT-qPCR was used to measure the relative expression of 88 genes involved in the wound healing process. On the 14th day, the number of cells stained with anti-CD68 and anti-CD31 antibodies was significantly larger in the tissues treated with 0.03% NAC compared with the control. Among the selected genes, 52 were upregulated and six were downregulated at different time points. Interestingly, NAC exerted a significant effect on the expression of 45 genes 60 days after its administration. In summation, a 0.03% NAC addition to the pre-incisional anesthetic solution improves neovasculature and increases the macrophages’ concentration at the wound site on the 14th day, as well as altering the expression of numerous genes that are responsible for the regenerative processes.

## 1. Introduction

Tissue regeneration in a wound environment occurs through an intricate interplay between keratinocytes, connective tissue and inflammatory cells [1]. The severity of inflammation is correlated with the extent of subsequent tissue scarring, a crucial aspect of wound regeneration—both visually and functionally [2]. The growth factors and cytokines play key roles in navigating the healing process [3]. Some of them, including platelet-derived, vascular endothelial and basic fibroblast growth factors, have been used to improve the tissue’s regenerative capacity in wounds [4]. Such interventions, however, are expensive and scarcely available, and some are associated with considerable side effects [5,6]. Thus, alternative substances that enhance the production of growth factors and key cytokines are needed.

N-acetylcysteine (NAC) is an antioxidative molecule that is capable of diminishing the number of free oxygen radicals and improving glutathione synthesis [7,8]. Apart from its cytoprotective properties, NAC was shown to improve tissue regeneration, acting via the phosphokinase C/Stat3 signaling pathway [9]. In rodent wound models, NAC enhanced healing, presumably by regulating the expression of growth factors and nitric oxide synthase, as well as by alleviating inflammatory processes [10,11].

Numerous cell types are involved in wound healing. A pivotal role is played by immune cells. The early phase of wound healing and the following shift to the repair phase is mainly mediated by neutrophils, which define the extent of tissue damage. Further, macrophages, with their dual phenotype (M1 or M2), mediate both pro- and anti-inflammatory actions during late inflammation, remodeling and scar maturation [12].

To date, no data exist on the effect of pre-incisional NAC administration on the created wounds and their healing process. Previous studies reported NAC’s effect on the wound environment, but only until the 14th day of healing. Moreover, we still do not know how NAC alters the gene expression profile of many growth factors and cytokines or inflammatory cells that are crucial for proper tissue regeneration.

In this study, we aimed to bridge these knowledge gaps by examining both the gene expression changes and presence of inflammatory cells in wounds exerted by pre-incisional NAC administration until the 60th day of healing. We hypothesized that, by reducing oxidative stress and the extent of the tissue damage, NAC may alleviate the early inflammation and affect wound healing molecular pathways. The study was performed as an extension of earlier histological observations [13].

## 2. Results

### 2.1. General Considerations

All animals completed the study. For convenience purposes, we named experimental groups according to the NAC concentrations used: NAC15 (0.015%), NAC30 (0.03%) and NAC45 (0.045%). In some cases, as a result of statistical analysis, we pooled the results of wounds pre-treated with NAC into one group: gNAC. Each analyzed section was tested (ANOVA or Kruskal–Wallis test) for statistically significant differences between the experimental groups (NAC15, NAC30 and NAC45). In the case of no difference between these groups, results were grouped—named gNAC—and compared with the control group (CONT).

### 2.2. Assessment of the Immunohistochemical Staining

**Figure 1:**
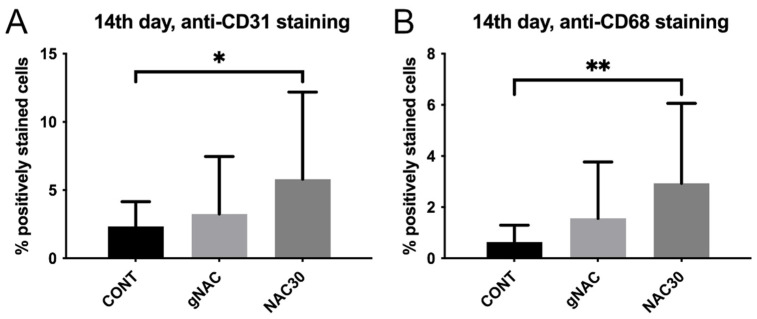
The graph represents differences in the percentage of positively stained cells with anti-CD31 (**A**) and anti-CD68 antibodies (**B**) between NAC30, gNAC and CONT on the 14th day. Data expressed as mean ± SD * *p* < 0.05, ** *p* < 0.01.

**Figure 2:**
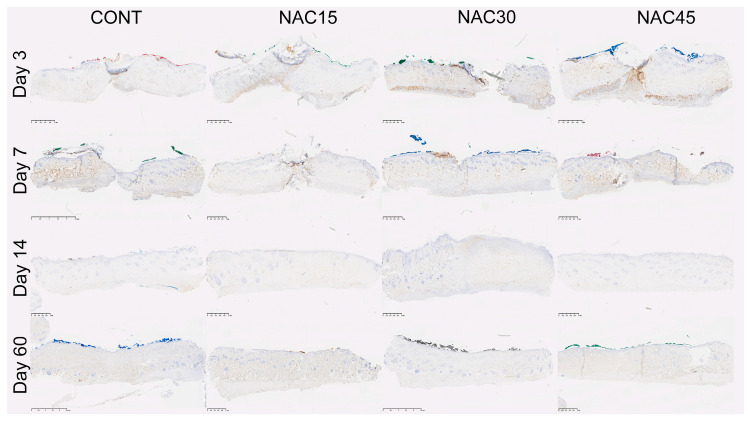
IHC staining for CD31 antigen. Representative samples from each group (NAC15, NAC30, NAC45 and CONT) at each harvesting time point.

**Figure 3:**
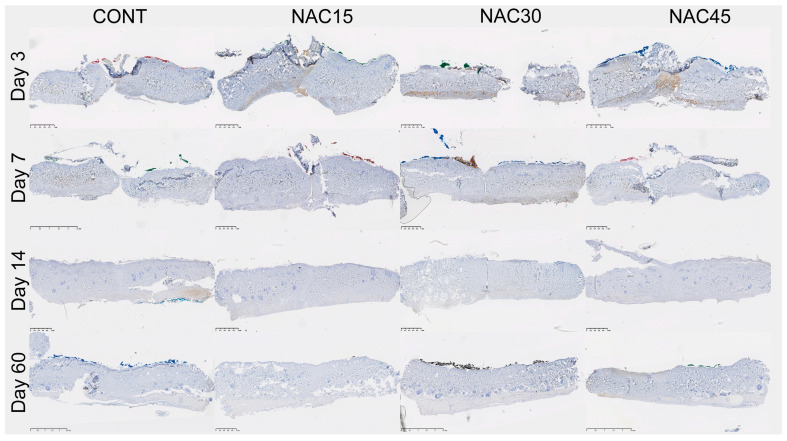
IHC staining for CD68 antigen. Representative samples from each group (NAC15, NAC30, NAC45 and CONT) at each harvesting time point.

**Figure 4:**
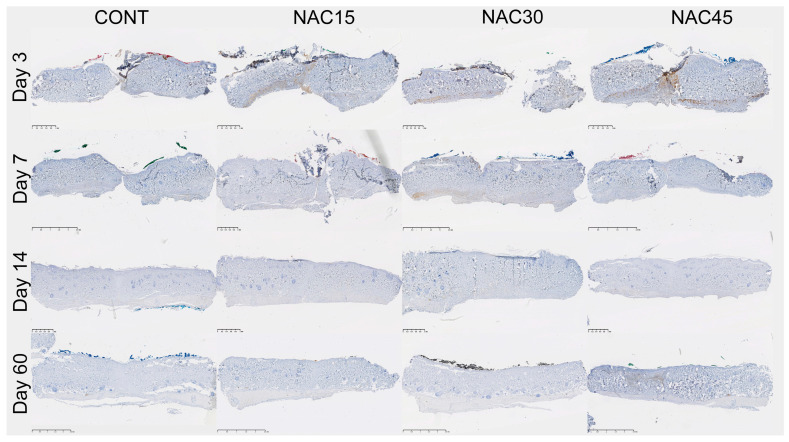
IHC staining for MPO. Representative samples from each group (NAC15, NAC30, NAC45 and CONT) at each harvesting time point.

There were no statistically significant differences among the NAC groups at the studied time points (ANOVA, *p* > 0.05), with one exception. On the 14th day, the mean values of the positive staining in the NAC30 group were different from the results in all of the other groups, including the control (*p* = 0.012). Thus, in the remaining time points, we compared the mean values of the studied parameters between the control and the gNAC group. Results from the 14th day, however, were further compared between the control, gNAC and the NAC30 groups (Table 1, Figure 1). Visual representations of each experimental group and control at four time points are shown in Figure 2, Figure 3 and Figure 4.

There were no statistically significant differences in the anti-CD31 antibody staining between the control and gNAC groups at any of the four time points (*p* > 0.05). We noted an increased expression of the CD31 antigen in the NAC30 group, in comparison with the control, on the 14th day (*p* = 0.04), illustrated by the percentage of positively stained cells, depicting the blood vessels density (Figure 1A). No differences were noted at any other time point. The mean values of all of the studied parameters decreased over time in all of the groups, with the peak values observed on the 3rd day.

There were no statistically significant differences in the anti-CD68 antibody staining between the control and gNAC groups at any of the four time points (*p* > 0.05). On the 14th day, there was a greater mean number of positively stained cells in the NAC30 group than in the control (*p* = 0.006) (Figure 1B).

Anti-MPO staining revealed no statistically significant differences in the neutrophils’ accumulation between the groups (*p* > 0.05). Additionally, the NAC30 group did not show statistically significant differences vs. CONT for both the percentage and the number of positively stained cells (*p* = 0.27, *p* = 0.21) on the 14th day. On the 3rd day, both parameters achieved the highest values within the observation period, whereas on the 7th and 14th day, they gradually decreased. There was an insignificant increase in anti-MPO-stained cells in both groups, versus the previous time point (14th day) (Table 1).

### 2.3. Genes’ Expression Analysis

**Figure 5:**
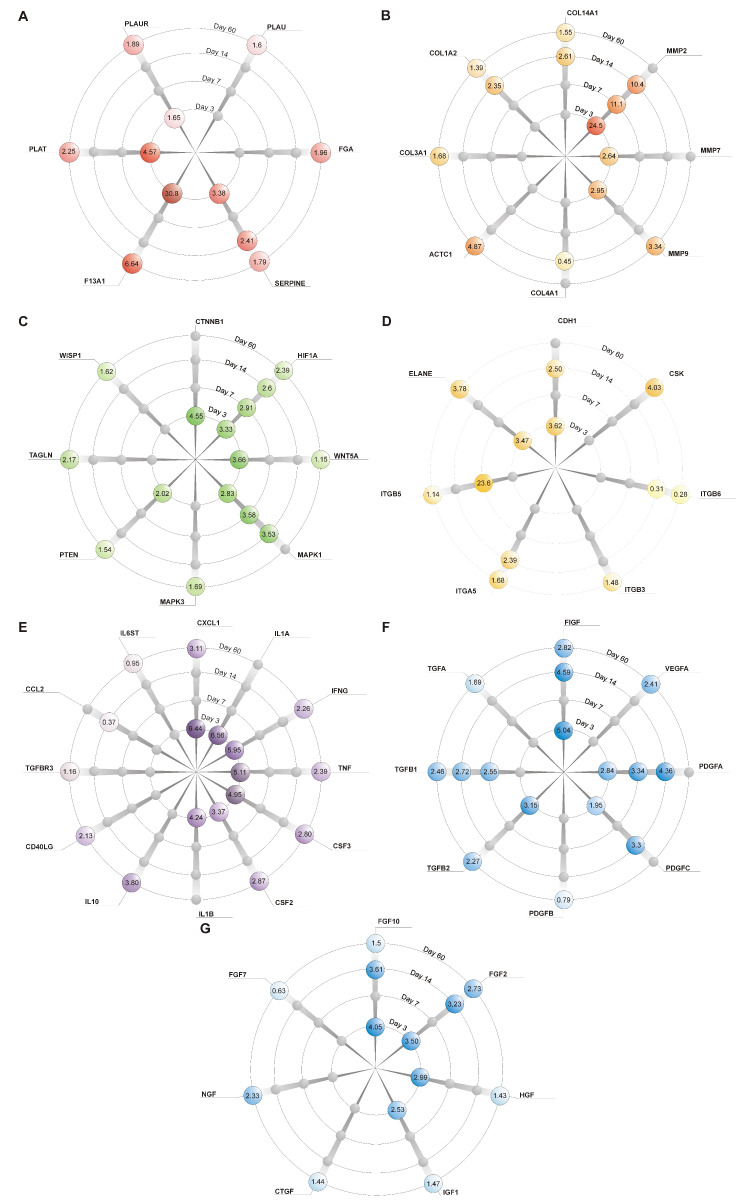
Changes in mRNAs expression on consecutive days of the experiment. Each radius represents one gene. Numbers in colored circles present differences in relative gene expression as compared with the control group. Values are presented as multiples of control expression levels (2^ΔΔcT^). Gray circles represent non-significant results (*p* < 0.05). Genes are divided into groups, which represent their molecular role in wound healing: (**A**) hemostasis and coagulation; (**B**) structural proteins, extracellular matrix components and remodeling; (**C**) proliferation, intracellular signaling and transcription factors; (**D**) cellular adhesion; (**E**) cytokines and chemokines; (**F**,**G**) growth factors.

We assessed the differences in the genes’ expression between the groups NAC15, 30 and 45 at the selected time points. We observed no significant differences in the expression of either the control or target genes (ANOVA, *p* > 0.05) between the experimental groups, and significant differences were observed when comparing them to CONT (ANOVA post-hoc Dunn tests, *p* < 0.05). Thus, we further investigated the overall effect of NAC on the studied genes’ expression. We analyzed the differences between the gNAC and CONT groups (Table 2, Figure 5).

We noted a significantly higher expression of 29 genes in the gNAC group, compared with CONT on the 3rd day. Fibrinogen XIIIa mRNA presented the most increased expression (30-fold). Among all of the other genes associated with hemostasis (at the wound site), we also observed an elevated expression of tissue plasminogen activator, an inhibitor of plasminogen activator and a urokinase receptor/plasminogen activator (4.57-, 3.38- and 1.65-fold, respectively) (Figure 5A). Tissue metalloproteinases, such as MMP2, MMP7 and MMP9, also showed a higher expression (Figure 5B). Cytokines, chemokines and proteins associated with inflammatory processes were also upregulated. Among the previously mentioned molecules, some presented a higher mRNA expression in the gNAC group: CXCL-1, IL-1A, IFN-γ, TNF, CSF3 and IL1B (>4-fold compared with CONT), as well as CSF2 (Figure 5E). An elevated expression of growth factors was confirmed in the NAC-treated tissues: FIGF, FGF10, FGF2, FGFβ2, HGF, PDGFA, IGF and PDCGF (Figure 5F,G). NAC administration, regardless of the concentration, caused an increase in mRNA expression in the case of β-catenin and WNT (4.55- and 3.66-fold, respectively), as well as MAPK1, PTEN and HIF1α (2.83-, 2.02- and 3.33-fold) (Figure 5C). Genes responsible for the adhesion processes, such as E-cadherin and neutrophil-produced elastase, also showed a higher expression in the gNAC group (3.62- and 3.47-fold, respectively). None of the chosen molecular targets showed a decreased expression in the gNAC group compared with the CONT on the 3rd day.

**Figure 6:**
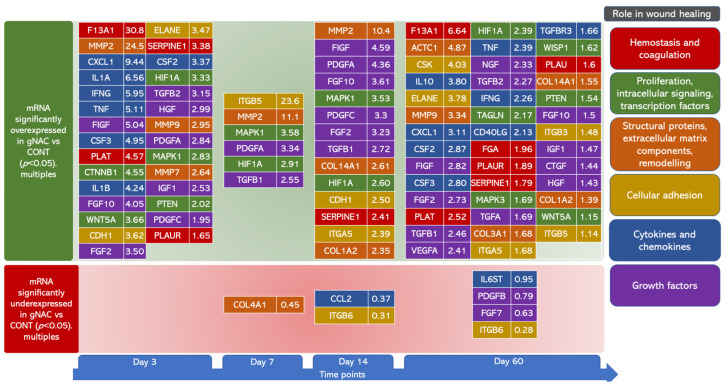
Gene expression matrix. The green part presents genes with a significantly higher expression on quantitative PCR analysis and the red part presents genes with a lower expression, when compared with control genes. Values are presented as multiples of control expression levels (2^ΔΔcT^). Different colors represent groups of genes that have a similar role in the wound healing process.

On the 7th day, the expression of seven genes varied between the gNAC and the CONT group. An elevated expression of mRNA was noted for adhesion-related genes integrin β5 (23.6-fold), tissue metalloproteinase MMP2 (11.1-fold) and growth factors PDGFA (3.34-fold) and TGFβ1 (2.55-fold). Moreover, a higher expression was observed for the signal transduction proteins MAPK1 and HIF1α (3.5- and 2.91-fold, respectively). Collagen IVA1 was the only gene whose expression was downregulated (by nearly two-fold) in the gNAC group compared with CONT. Furthermore, CCL2 chemokine expression was decreased 2.7-fold but was not statistically significantly (*p* = 0.05) (Figure 6).

On the 14th day, MMP2 expression was still increased (10.4-fold) (Figure 5B). In addition, a significantly elevated mRNA expression was observed for growth factors FIGF, PDGFA, FGF10, PDGFC, FGF2 and TGFβ1 (4.59- to 2.71-fold) (Figure 5G,F). The expressions of MAPK1 and HIF1α were still higher in the gNAC group compared with that of CONT (3.53-fold and 2.6-fold, respectively). An increased expression of two forms of collagen was noted: collagen XIVA1 and IA2 (2.61- and 2.35-fold). It was accompanied by an increase in E-cadherin and integrin α_5_ expression (2.5- and 2.4-fold) (Figure 5D). Moreover, mRNA expression for the inhibitor of the plasminogen activator was higher in gNAC vs. CONT (2.42-fold). A decreased expression of chemokine CCL2 (2.7-fold) and integrin β_6_ (3-fold) was observed in the gNAC compared with CONT.

On the 60th day, a higher expression of 41 types of mRNA in the gNAC group was observed. Among the hemostasis-associated genes, F13A1, PLAT, FGA, PLAUR and SERPINE1 presented an elevated expression (6- to 1.6-fold) (Figure 5A). Moreover, the growth factors’ expression was also upregulated at that time: FIGF, FGF2, TGFβ1, VEGFA, TNF, NGF, TGFβ2, TGFA, FGF10, IGF1, CTGF and HGF (Figure 5G,F). Their expression levels were lower than at previous time points, ranging from a 1.43- to 2.82-fold increase compared with CONT. Similarly, an elevated expression of cytokines and chemokines mRNAs was noted on the 60th day—especially CSF2, CSF3, IFNγ and TGFβ3 (2.87- to 1.66-fold) (Figure 5E). At this time, a higher expression was shown in the case of genes associated with structural proteins, the extracellular matrix and remodelling: actin-α, MMP9 (4.87- and 3.34-fold) and collagens IIIA1, XIVA1 and IA2 (1.68- to 1.39-fold). (Figure 5B) Moreover, an elevated expression of CSK and ELANE (4.87- and 3.34-fold), as well as integrin α5, β3 and β5 (1.68- to 1.14-fold) (Figure 5D), was also noted. The anti-inflammatory IL-10 expression was upregulated (3.8-fold), similarly to CXCL1 (3.11-fold) and ligand CD40 (2.13-fold). Among the signal transduction-associated genes, the expression levels of HIF1α, TAGLN, MAPK3, WISP, PTEN and WNT5A were higher (2.39- to 1.15-fold) in the gNAC group (Figure 5C). A lowered mRNA expression was noted for four genes: IL6ST, growth factors PDGFB and FGF7 (1.26- and 1.58-fold) and integrin β6 (3.57-fold) (Figure 6).

## 3. Discussion

The study presents a molecular follow-up of our previous experiment [13]. Briefly, we showed that a single pre-incisional dose of NAC (especially 0.03%) significantly decreased the scar area and width at an early wound healing stage. We noted no explicit histological features, such as an immense inflammatory cell influx, tissue destruction, scant or excessive angiogenesis or abundant collagen fiber deposition. A quantitative analysis of collagen fiber organization [14] showed increased dynamics in collagen fiber orientation on the 7th and 14th days in the scars and surrounding tissues.

Here, we measured the relative expression of 88 genes involved in wound healing. We found that a single dose of NAC upregulates 52 and downregulates six of them. Importantly, this influence is visible for up to 60 days after NAC administration.

We showed that NAC increases the amount of mRNA of certain collagen forms on days 14 and 60. It also decreases the expression of α protein of collagen IV by two-fold on the 7th day. Collagen IV is present in the basement membrane and may take part in re-epithelialization [15,16]. A decrease in its expression may be unfavorable; however, no disturbance in the epithelialization or wound closure in the gNAC group was noted. This observation corresponds with the lowered variance of the collagen fibers arrangement at the peri-wound site on the 7th day [13]. On the other hand, we observed an elevated expression of several types of collagens on days 14 and 60. In scar sections stained with Masson’s trichrome, we observed a lower variance of collagen fibers in the surrounding tissue on the 7th day, but a less organized fiber variance in the scar itself on the 14th day. This suggests increased dynamics of scar remodeling. It was followed by an enhanced expression of collagen IA2, which is abundantly present in the skin, contributes to a faster closure of the wound and may be beneficial [17]. The upregulated expression of collagen XIV α1 mRNA is also favorable since its deficiency is frequently noted in slowly healing diabetic wounds [18]. Therefore, research investigating the use of NAC in diabetic patients might be worth considering. A higher expression (>two-fold) of collagen mRNA on the 14th day may be associated with its increased arrangement variance at the scar site. In addition, collagen expression was elevated even on the 60th day in the gNAC group, suggesting that the wound/scar tissue underwent remodeling throughout the whole observation period. At the same time point (60th day), the upregulation of collagen III expression was noted. This collagen type contributes to the myofibroblasts’ differentiation and scar remodeling [19]. An increased expression of this gene may intensify the wound contraction. Mice with collagen III knockout present larger mean areas of the wounds [20]. The myofibroblasts’ differentiation may have also been promoted by an elevated expression of transgelin [21].

In order to explore the influence of NAC on inflammatory cell influx, IHC staining was performed. Antigens CD68 and MPO, which are characteristic for macrophages and neutrophils, respectively, were detected. We showed that a 0.03% NAC administration was associated with a higher expression of CD68 on the 14th day. At that time, various processes led to the resolution of the inflammation and promotion of neoangiogenesis, and the formation and/or remodeling of the tissue structural elements [1]. An upregulated expression of CD68 on the 14th day may have been caused by the migration of monocytes and their transformation into tissue macrophages. These cells play a major role in scar remodeling [22,23], releasing tissue metalloproteinases, including MMP2 [24]. Its expression was also upregulated at that time.

A lack of differences in the anti-MPO staining suggests that NAC administration does not influence the number of neutrophils migrating to the wound site. This corresponds with the subjective histological assessment results, which showed no significant differences in the severity of inflammation (acute or chronic) between the groups [13].

It seems that a single pre-incisional NAC injection does not have a significant impact on the populations of cells taking part in the inflammatory response at the wound site. Nonetheless, NAC modulates both the course of inflammation and subsequent stages of wound healing by increasing the expression of numerous cytokines, chemokines and growth factors. On the 3rd day, CXCL1 was one of the most highly expressed chemokines. Its function is to recruit and activate neutrophils and macrophages in a response to hypoxia and Il-1 or TNF [25,26]. Expressions of both of the previously mentioned cytokines were also upregulated on the 3rd day after NAC administration. TNF modulates the function of macrophages and enhances the scar’s mechanical resilience [27]. However, on the 60th day, it was counteracted by the WISP protein [28]. IL-1α stimulates the expression of growth factors (FGF and EGF) and enhances the proliferation of both keratinocytes and fibroblasts [29]. Similarly, IL-1β stimulates fibroblasts to produce collagen [30]. Other cytokines, such as IFN-γ, CSF2 and CSF3, showed an increased expression on the 3rd day and had a beneficial effect on the wound healing process [31,32,33]. At the later time points, NAC did not increase the expression of inflammatory cytokines. On the 7th and 14th days, CCL2 expression was reduced 2.7-fold (*p* = 0.05 and *p* = 0.02). CCL2 recruits neutrophils and macrophages to the wound bed. At the stage of remodeling, a massive inflammatory response could have an adverse effect on scar formation [25,34,35]. Thus, it suggests the beneficial influence of NAC.

On the 60th day, a substantially increased expression of an anti-inflammatory cytokine IL-10 was noticed. Il-10 may contribute to inhibiting the excessive remodeling and fibrosis of the mature scar [36]. An elevated expression of CSF-2 and -3 may stimulate angiogenesis, which is reflected by the higher expression of VEGF. An increased expression of IFN-γ at later stages of wound healing may be beneficial, since it prevents excessive fibrosis and collagen production [37]. It may also antagonize a higher expression of TGF-β1, which promotes fibrosis and potentiates inflammatory processes at the wound site [38,39]. The expression of ligand CD40 is also increased, which leads to the stimulation of wound remodeling via MAPK and MMP2 activation [40].

Extensive inflammatory processes (mediated by chemokines or immune cells) negatively affect wound healing. However, the same processes also promote tissue regeneration at further stages of wound healing [41]. For instance, inhibiting the pro-inflammatory IL-1β pathway increases the expression of other inflammatory cytokines and downregulates the growth factor expression [42]. In our study, IL-1β upregulation (3rd day) was not followed by the excessive influx of inflammatory cells—a finding corroborated by other studies [43].

Other researchers showed that NAC promoted neo-angiogenesis, which enhanced the healing process [44], especially in the case of diabetic wounds in animal models [10]. We observed that the CD31 antigen expression was significantly increased on the 14th day in the group treated with 0.03% NAC. Furthermore, an elevated expression of vascular endothelial growth factor (VEGF) was noted on days 3, 14 and 60 (5.4-, 4.59- and 2.82-fold, respectively, and VEGFA increased 2.4-fold on the 60th day). A higher expression of VEGF after NAC administration was also noted in other studies [10]. Apart from its role in the new vessels’ formation, VEGF also regulates the wound remodeling, and a persistent overexpression of this factor may lead to excessive fibrosis and keloid formation [45]. At all of the studied time points, an increased expression of HIF1α was observed. This transcription factor promotes angiogenesis and plays a vital role in wound healing via upregulating the expression of metabolic proteins (GLUT-1), integrins, cytokines, growth factors (TGF-β and VEGF) and elements of the extracellular matrix (collagen type I, fibronectin). Moreover, it influences the wound remodeling process [46]. An elevated expression of mRNA of many of the above-mentioned molecules was observed at all of the studied time points. HIF1α has a beneficial effect on wound healing, and interventions aiming to increase its expression resulted in the improvement of the wound healing processes, especially in the ischemic model [46,47]. NAC protects neurons against ischemia via HIF1 α overexpression in a rodent model of ischemic stroke [48]. We also observed that a single intradermal NAC injection continually upregulates the expression of HIF1α for 60 days. Other pro-angiogenic factors that showed increased expressions in the NAC-treated group were CSF2 and CSF3 (on the 3rd and 60th day) [32,33]. Despite an elevated expression of the previously mentioned factors, an intensified neo-angiogenesis has not been confirmed by the histological assessment [13].

We showed that a single intradermal NAC administration altered the expression of numerous genes for up to 60 days. It is especially evident in the case of cytokines, proteins acting via signal transduction pathways and extracellular matrix proteins. Moreover, an elevated expression of factors associated with hemostasis was observed—fibrinogen XIII (more than 30-fold), tissue plasminogen activator, plasminogen activator/urokinase receptor and plasminogen activator inhibitor—in NAC-treated samples. Wound healing is impaired in the absence of F13A1 and PLAT or in the case of the malfunction of these factors [49,50]. SERP1NE1 and PLAUR promote a transition into the inflammatory phase of wound healing [51,52]. Increased amounts of the mRNA of these genes on the 60th day were associated with the promotion of wound remodeling. An increased expression of fibrin confirms these assumptions—fibrin modulates the intercellular interactions during remodeling, binds FGF2 and VEGF and stimulates cellular proliferation, acting via integrins and non-integrin receptors (e.g., I-CAM) [53]. At all of the time points, a higher expression of growth factors was noted. C-fos-induced growth factor, i.e., VEGD, not only promotes angiogenesis but also stimulates the deposition of collagen fibers, neo-epithelialization and lymphatic vessel formation [54,55]. FGF-10 and FGF-2 mediate the proper healing in both fetal and adult life, and their knock-out implies the impairment of this process [56,57]. FGF-2 has an additional effect where it interacts with the cytoskeleton via the ACTC1 gene; its expression was also elevated on the 60th day [58]. HGF enhances the neo-epithelialization via the β1-integrin/ILK pathway [59], and its exogenous administration improves the wound healing in diabetes mellitus in mice [60]. PDGFA stimulates macrophages to participate in the mature scar remodeling and, at earlier stages, improves neo-epithelialization [61,62]. PDGFC boosts the wound healing process in a similar way [63]. IGF-1 stimulates fibroblasts and keratinocytes to proliferate and induces the expression of HIF1α [64,65,66]. On the 60th day, connective tissue growth factors may induce the production of collagen fibers and fibroblast proliferation, acting via the SMAD pathway [67,68]. On the other hand, NGF boosts the migration of fibroblasts, mediated by the PI3K/Akt-Rac1-JNK and ERK pathways [69].

The interaction between the cells and the extracellular matrix and its modifications are important components of the wound healing process. We noted a higher expression of tissue metalloproteinases in the gNAC group compared with the CONT at numerous time points. The expression of MMP2 was particularly increased. Its role is to degrade collagen IV, which is one of the components of the basement membranes. The metalloproteinases’ activity is desired at the early stages of wound healing, since they help to dispose of the damaged tissue elements. Meanwhile, along with the overexpression of MMP2, we observed increased dynamics of collagen fiber variation surrounding the scar or in the scar itself [13]. However, a persistent overexpression of these enzymes may also impair the regenerative process [70,71]. They take part in an active extracellular matrix remodeling, thus the presence of MMP9 on the 60th day is understandable. As MMP9 degrades the extracellular matrix, a subsequent release of cytokines follows, influencing the process of angiogenesis [72]. Moreover, among all of the metalloproteinases, a deficit of MMP7 is the most severe, causing a major wound healing disorder [73].

NAC administration also influenced the expression of the mRNA of the proteins associated with adhesion and interaction processes. We noted a higher expression of E-cadherin. Its role is to maintain intercellular adhesion, cell migration and the division of the epithelial cells [74], playing a vital role in re-epithelialization [75]. Cell migration may have also been promoted by the serine kinase, as its expression was upregulated on the 60th day [76]. On days 3 and 60, an elevated expression of neutrophil-produced elastase was observed—during the inflammatory phase, it has an antibacterial effect; however, at later stages, it takes part in the mature scar remodeling [71,77]. NAC-treated tissues showed higher expressions of integrin-β_5_, α _5_ and β_3_, and a lowered expression of β_6_ from the 7th day. Overexpressed integrins are the key receptors for angiogenesis (α_5_), or have no significant role in the healing process (β_3_, β_5_) [78,79]. A decreased expression of integrin β_6_ on days 14 and 60 indicates that the epithelium remodeling process is less intensive [80]. At that time, most of the scars had a properly formed epithelial layer [13].

The transforming growth factor β (TGFβ) family is an important group of regulatory proteins, influencing the processes of wound healing in a pleiotropic fashion. NAC administration resulted in an increased expression of different isoforms of TGF at numerous time points. TGFs promote the inflammatory cells’ recruitment and initiate the formation of the granulation tissue, acting via the SMAD pathway at the early stages of healing [81,82]. A decreased expression of these factors in diabetic wounds causes impaired wound healing. The exogenous administration of TGFβ2 improves the process significantly [83,84]. This finding supports the potential usefulness of NAC administration in wound treatment in diabetic patients. An elevated expression of TGFβR3 is associated with scar remodeling and stimulates cells migration [85]. Thus, NAC may have acted on wound healing via the TGFβ pathway.

In this experimental setting, NAC modulated numerous cellular pathways. It not only had a wide upregulating effect on the expression of many cytokines and growth factors but also increased the expression of the signal transduction pathways’ components. As described before, NAC significantly upregulated the expression of transcription factor HIF1α. We also noted an elevated expression of the WNT/β-catenin pathway proteins (4.5- and 3.6-fold) already on the 3rd day. These molecules promote cellular proliferation, migration and extracellular matrix remodeling at every stage of wound healing via the activation of the proteins c-Myc and Hes1 [86,87]. It corresponded with the macroscopic processes occurring on days 3 and 60, when the expression of these factors was increased [13]. NAC also influenced the expression of the mRNA of MAPK. MAPK1 enhanced wound healing through the stabilization of the extracellular matrix remodeling, stimulating both the epithelial cell migration and overall epithelialization on days 3, 7 and 14 [88]. Interestingly, it may become a molecular target for wound healing improvement with the use of vemurafenib [89]. MAPK3, on the other hand, promoted angiogenesis via the stimulation of the expression of VEGF on the 60th day [90]. An overexpression of the mRNA of PTEN was noted at an early stage of healing. This phosphatase takes part in wound healing, regulating the proliferation, migration and survival of the cells [91].

NAC is a small and relatively simple molecule. It was proved to act as a free radical scavenger and a cytoprotective agent [92,93]. Numerous authors reported that NAC decreases the load of oxidative stress in healing tissues. Further, the tissue damage and ROS levels regulate the extent of the immunological response and cellular influx to the wound bed [94]. Early stages of wound healing incorporate neutrophil migration, which provides clearance of the wound from bacteria and dead cells [94]. Thus, low levels of ROS are required for the proper initiation of wound healing [94] and their excess contributes to impaired healing [95,96]

Thus, NAC-mediated wound healing was intended to act via preventing the excessive tissue damage and oxidative stress level. Other authors showed decreased ROS in the healing tissue after NAC administration [10,97,98]. In our study, we have not observed the impact of the decreased influx of inflammatory cells—on both the histological [13] and immunohistochemical analysis. However, we reported numerous aforementioned alterations in the gene expression at each timepoint of the wound healing. Taking into consideration the profile of their expression, some targets appear to be the most important in the promotion of the wound healing. Namely, HIF1α showed a continuous overexpression at all time points. The molecule transcriptionally upregulates the expression of many other genes that enhance the wound repair, such as metabolic proteins, integrins, growth factors and ECM components. Many of them were upregulated in our study (ITGB6, ITGB3, ITGA5, MMP2, MMP9, FGFs, IGFs, VEGFA and TGFs). HIF1α-related MMP2 and MMP9 overexpression were reported by Tsai et al. [72]; MMP2 was especially highly overexpressed in our case. Furthermore, HIF1α regulates appropriate inflammatory and angiogenic responses in normal skin wound healing [46]. Such a phenomenon was reflected in our result with the increased expression of IL1α, CXCL1, TNF, CSF2, CSF3, IFNγ, TGFβ s and FIGF, VEGFA or PDGFC. In addition, we observed the upregulation of TGFβ1 in several time points, which also induces HIF1α [82,99]. Sanchez at al. also suggested that overexpressed TGFβ1 and HIF1α have a synergistic effect on neovascularization via VEGF, which corresponds with our findings [100].

NAC has been proven to induce HIF1α overexpression in other studies. Zhang et al. limited the stroke impairment of the murine brain by NAC pretreatment. NAC acted via HIF1α and its downstream targets (EPO, GLUT3) [48]. This was preceded with an in vitro study that confirmed that NAC induced HIFα overexpression [101]. Moreover, the impaired expression of HIF1α or its knock-outs contributes to impaired wound healing, especially in the case of diabetic wounds [46,102]. Despite these observations, further studies are needed to confirm if HIF1α comprise a major axis of NAC-mediated wound healing enhancement.

Along with HIF1α, MAPK1 exhibited long-lasting overexpression. The latter is a key factor orchestrating cellular proliferation, migration and differentiation [103]. All of these processes are crucial to normal wound healing. Importantly, this molecule was significantly upregulated on the 7th day, which represents the proliferative phase of tissue regeneration. Another key molecule that showed an increased expression at that time was TGFβ. It has been shown to stimulate fibroblast proliferation, collagen synthesis and deposition (possibly potentiating MAPK/ERK proliferative actions). This growth factor also stimulates angiogenesis, similarly to the previously described HIFα [82]. Although the methods utilized in this study do not allow to claim causality, it is tempting to hypothesize that the increased activity of key growth and transcription factors at early stages of wound healing prompted faster tissue regeneration after NAC administration.

A beneficial influence of NAC on wound healing in this model is consistent with the observations made by the authors investigating the role of this molecule in wound healing in different settings, e.g., in the case of burns or excisional models [9,10,44,97,98,104]. The discrepancies, such as a non-significant inflammatory process alleviation or merely subtle changes in the histological assessment, may be caused by differences in the experiment model, the carrying out of a single NAC intradermal injection—a route of administration not previously described—or a simultaneous injection of lidocaine and adrenaline [13]. These substances affect the wound healing itself, as well as the distribution of a locally administered agent [105].

## 4. Materials and Methods

### 4.1. Animals and Surgical Procedure

A detailed description of the surgical procedure is available in the histological report [13]. Briefly, the experiments were approved by the First Local Ethics Committee in Warsaw (Protocol no 304/2017). A total of 24 male Sprague–Dawley rats underwent intradermal preincisional injection of either control solution of local anesthetic (0.5% lidocaine (Lignocainum Hydrochloricum WZF, Polfa Warszawa, Poland)) + vasoconstrictor (1: 100.000 epinephrine (Adrenalina WZF, Polfa Warszawa, Poland) or one of three concentrations of NAC (Acetylcysteine, Sandoz and Poland): 0.015%—NAC15 group, 0.03%—NAC30 group and 0.045%—NAC45 group, dissolved in anesthetic solution as in control group (0.5% lidocaine with 1:100.000 epinephrine in sterile water). Each rat had six 1.5cm long incisions made—3 on each side the of the rat’s back. A randomly chosen side (3 sites) was injected with the control solution, and the others were injected with NAC solutions. (1 concentration/site).

### 4.2. Scar Tissue Collection and Immunohistochemical Analysis

On the 3rd, 7th, 14th and 60th day after the operation, six rats were sacrificed. Tissue harvesting time points were chosen in order to observe consecutive phases of wound healing (3rd day—inflammatory phase, 7th day—proliferative phase, 14th day—early remodeling phase and 60th day—advanced remodeling phase).

The central part of the scar was preserved in 10% formalin solution for histological and immunohistochemistry staining. Samples were cut on a microtome in order to obtain 3–5-micron sections. After standard deparaffinization protocol, immunohistochemistry staining was performed with EnVision™ FLEX Mini Kit, High pH (DAKO, Agilent, Santa Clara, CA, USA) to assess the presence of immune cells. Following primary antibodies were used: anti-CD68 (ED-1) clone KP1, IgG1κ, (IR609, DAKO, Agilent, Santa Clara, CA, USA) for tissue macrophage visualization, anti-MPO (myeloperoxidase) IgG1κ (IR511, DAKO, Agilent, USA) for neutrophils migration marking and anti-CD31 clone JC70a, IgG1κ (IR610, DAKO, Agilent, Santa Clara, CA, USA) for blood vessel identification [106]. For positive control, we used the rat’s spleen (also inbred, male Sprague–Dawley rat). Immunohistochemical staining was performed using Autostainer Link48 (DAKO, Agilent, Santa Clara, CA, USA). Staining procedures were carried out according to the manufacturer’s guidelines. All stained sections were scanned at 40× magnification with NanoZoomer XR C9600-12 (Hamamatsu, Iwata City, Japan) in order to achieve WSI scans (Whole Slide Images).

WSI underwent automated analysis in QuPath [107]. Uploaded files were manually checked for artifacts (stain traces, blood clots, folded tissue, etc.) and ROI (region of interest) was outlined. Protocols for analysis of each staining were optimized and are attached in Supplementary File 1. Briefly, nuclei identification was optimized, further types of IHC staining locations (intra- or extracellular) were chosen and optimized. Results were presented as % of positively stained cells and the absolute numbers of stained cells per 1 mm^2^ of tissue.

### 4.3. Gene Expression Analysis

The rostral part of each scar was preserved in RNAlater according to manufacturer’s guidelines (Sigma, Germany). Further, samples were thawed, and an 8 mm punch biopsy was taken out of each scar’s center. RNA was isolated with a modified Chomczyński method (Trireagent, Thermo Fisher Scientific, Waltham, MA, USA) [108]. Biopsies were poured with 200 μL Trireagent solution and homogenized mechanically with metal beads (Beads, Tissue Lyser, Thermo Fisher Scientific, Waltham, MA, USA) for 3 min. Further, we followed the original manufacturer’s protocol. RNA was suspended in 60 μL of DNAse/RNAse-free water and stored in −80 °C. Total RNA quality and quantity were checked with NanoDrop (NanoDrop 2000/c. Thermo Fisher Scientific, Waltham, MA, USA, the cutoff for downstream analyses: A260:A280 > 1.8 and A260:A230 > 1.8). Reverse transcription was performed with PrimeScriptTM RT Master Mix (Takara) on 500 ng of RNA input. cDNA libraries were stored until further reactions in −20 °C.

qPCR (quantitative polymerase chain reaction) assay included 88 targets related to the wound healing process and 6 endogenous controls: β-actin, GAPDH, LDHA, NONO, PPIH and GDC (for target list and primer sequences, see Supplementary Table S1). Primers derived from published articles were related to wound healing or were designed in Primer-Blast (NCBI). All primers were tested for optimal concentration (50, 300 or 900 nM). For qPCR, we used PowerUp™ SYBR^®^ Green Master Mix (Thermo Fisher Scientific, Waltham, MA, USA) and performed the assay according to manufacturer’s guidelines. Reactions were performed in 384-well plates in the total volume of 3 μL. Each sample was analyzed in technical quadruplicate. Reactions were run on Viia 7 (Life Technologies, Thermo Fisher Scientific, Waltham, MA, USA). ΔΔct was calculated for each target in NAC wound versus control within the same animal. Final results were presented as mean multiples of control expression levels with standard deviations [109].

### 4.4. Statistical Analysis

Data distribution was verified with the Shapiro–Wilk test. Further, two-tailed ANOVA with posthoc Dunn test, Student’s t-test and Mann–Whitney U tests were used. Statistical analyses were performed in Statistica 13 (StatSoft Inc., Dell Statistica, Tulsa, OK, USA) and plots were designed in GraphPad Prism 9.1 (GraphPad Prism, San Diego, CA, USA) with the threshold of statistical significance set at *p* ≤ 0.05.

## 5. Conclusions

A pre-incisional intradermal NAC injection is associated with multiple molecular changes that are mediated by a significant number of growth factors and cytokines. Eventually, an increased number of blood vessels and macrophages on the 14th day was observed. These outcomes require further research concerning the exploration of both the mechanism driving the NAC-promoted wound healing and the multiple dose administration efficiency. 

## Figures and Tables

**Table 1:** Data gathered on immunohistochemical staining with anti-CD-31, anti-CD68 and anti-MPO antibodies at all time points. Data expressed as mean ± SD. Bolded results are statistically significant.

	Day3			Day 7			Day 14					Day 60		
	CONT	gNAC	*p*	CONT	gNAC	*p*	CONT	gNAC	*p*	NAC30	*p*	CONT	gNAC	*p*
	Mean ± SD	Mean ± SD		Mean ± SD	Mean ± SD		Mean ± SD	Mean ± SD		Mean ± SD		Mean ± SD	Mean ± SD	
CD31% of positive cells	8.92 ± 4.49	10.5 ± 7.23	0.42	6.24 ± 3.92	5.22 ± 3.01	0.38	2.34 ± 1.81	3.25 ± 4.22	0.40	5.81 ± 6.37	**0.04**	3.08 ± 2.99	2.36 ± 1.27	0.88
CD31 *n* of positive cells/mm^2^	179.58 ± 103.56	207.89 ± 129.16	0.47	135.09 ± 99.88	112.67 ± 76.35	0.45	41.182 ± 38.698	58.362 ± 79.914	0.41	105.51 ± 120.66	**0.05**	62.277 ± 70.29	46.962 ± 29.53	0.76
CD68% of positive cells	9.14 ± 5.06	9.51 ± 4.44	0.81	4.30 ± 3.29	3.52 ± 2.54	0.43	0.64 ± 0.65	1.56 ± 2.21	0.42	2.94 ± 3.12	**0.006**	1.39 ± 1.27	1.50 ± 1.75	0.82
CD68 *n* of positive cells/mm^2^	334.75 ± 182.45	353.34 ± 175.26	0.75	164.72 ± 128.46	131.10 ± 90.12	0.37	23.003 ± 26.45	55.603 ± 80.79	0.46	106.04 ± 115.75	**0.008**	45.854 ± 43.33	47.069 ± 53.14	0.94
MPO% of positive cells	8.87 ± 4.83	8.84 ± 4.76	0.98	2.76 ± 1.61	2.88 ± 1.63	0.82	0.69 ± 0.40	0.88 ± 0.78	0.96	1.01 ± 1.02	0.27	1.81 ± 1.52	1.52 ± 1.11	0.48
MPO *n* of positive cells/mm^2^	303.38 ± 192.10	322.24 ± 195.06	0.77	102.35 ± 63.24	100.76 ± 60.13	0.93	21.342 ± 13.62	28.009 ± 26.41	0.98	34.230 ± 37.23	0.21	51.286 ± 39.32	43.775 ± 33.28	0.54

**Table 2:** Differences in mRNAs expression at each time point obtained from quantitative PCR analysis results. Values are presented as multiples of expression levels in the control group (2^ΔΔcT^).

Folds of Expression Versus CONT (2^ΔΔ^^cT^)												
	Day 3			Day 7			Day 14			Day 60		
	CONT	gNAC		CONT	gNAC		CONT	gNAC		CONT	gNAC	
	Mean ± SD	Mean ± SD	*p*	Mean ± SD	Mean ± SD	*p*	Mean ± SD	Mean ± SD	*p*	Mean ± SD	Mean ± SD	*p*
ACTC1	1 ± 1.00	1.35 ± 1.50	0.44	1 ± 2.11	4.15 ± 2.29	0.09	1 ± 1.14	1.78 ± 1.53	0.21	1 ± 0.65	4.87 ± 2.27	0.00
ACTA2	1 ± 2.79	1.74 ± 1.35	0.67	1 ± 0.66	0.98 ± 0.99	0.96	1 ± 0.67	0.69 ± 1.65	0.30	1 ± 0.38	0.51 ± 2.82	0.89
ANGPT1	1 ± 3.80	2.29 ± 1.94	0.48	1 ± 2.53	1.11 ± 2.41	0.91	1 ± 2.85	0.82 ± 3.09	0.86	1 ± 1.35	0.31 ± 1.99	0.86
COL14A1	1 ± 0.36	1.07 ± 1.44	0.78	1 ± 0.91	1.84 ± 1.80	0.15	1 ± 0.92	2.61 ± 2.06	0.04	1 ± 0.48	1.55 ± 1.55	0.02
COL1A2	1 ± 0.80	1.37 ± 0.98	0.28	1 ± 1.04	1.68 ± 1.69	0.25	1 ± 0.41	2.35 ± 2.04	0.02	1 ± 0.45	1.39 ± 2.05	0.02
COL3A1	1 ± 0.67	1.49 ± 1.09	0.16	1 ± 0.84	1.87 ± 2.55	0.22	1 ± 0.98	2.40 ± 2.29	0.43	1 ± 0.70	1.68 ± 1.97	0.01
COL4A1	1 ± 0.46	1.32 ± 0.83	0.15	1 ± 0.64	0.45 ± 1.52	0.02	1 ± 0.51	0.86 ± 1.12	0.56	1 ± 0.30	0.65 ± 1.98	0.33
COL4A3	-	-	-	-	-	-	-	-	-	-	-	-
COL5A1	1 ± 0.68	1.13 ± 1.41	0.69	1 ± 0.61	0.76 ± 2.40	0.56	1 ± 0.67	1.57 ± 1.70	0.77	1 ± 0.49	1.12 ± 1.05	0.71
COL5A3	1 ± 3.34	3.77 ± 1.05	0.34	1 ± 1.16	1.18 ± 1.13	0.69	1 ± 0.68	0.90 ± 1.38	0.75	1 ± 0.61	0.78 ± 1.61	0.28
CCL2	1 ± 0.77	1.45 ± 1.96	0.35	1 ± 1.21	0.36 ± 1.57	0.05	1 ± 0.79	0.37 ± 1.77	0.02	1 ± 0.80	0.82 ± 1.72	0.06
CD40LG	1 ± 1.16	1.50 ± 1.28	0.32	1 ± 1.28	1.72 ± 2.38	0.34	1 ± 1.02	1.24 ± 2.28	0.66	1 ± 1.14	2.13 ± 1.77	0.04
CDC42	1 ± 6.02	0.09 ± 1.77	0.25	1 ± 4.61	1.92 ± 2.87	0.68	1 ± 4.07	0.10 ± 2.19	0.11	1 ± 1.21	2.41 ± 2.27	0.23
CDH1	1 ± 1.02	3.62 ± 1.04	0.00	1 ± 0.48	2.23 ± 1.64	0.15	1 ± 0.49	2.50 ± 2.11	0.02	1 ± 0.16	2.49 ± 2.04	2.01
CSF2	1 ± 1.02	3.37 ± 2.09	0.01	1 ± 1.34	1.80 ± 2.78	0.35	1 ± 1.47	1.10 ± 1.83	0.85	1 ± 1.24	2.87 ± 1.96	0.04
CSF3	1 ± 0.86	4.95 ± 2.40	0.02	1 ± 1.37	2.65 ± 2.94	0.15	1 ± 1.26	1.31 ± 2.85	0.66	1 ± 0.60	2.80 ± 1.91	0.01
CSK	1 ± 1.07	1.32 ± 1.04	0.44	1 ± 2.95	1.29 ± 3.16	0.81	1 ± 0.32	2.23 ± 2.71	0.58	1 ± 1.12	4.03 ± 1.42	0.01
CTGF	1 ± 1.89	2.22 ± 1.41	0.21	1 ± 0.80	1.16 ± 1.40	0.65	1 ± 0.91	2.14 ± 1.68	0.07	1 ± 0.33	1.44 ± 1.59	0.01
CTNNB1	1 ± 2.69	4.55 ± 1.12	0.00	1 ± 0.83	0.59 ± 1.62	0.18	1 ± 0.94	0.73 ± 2.72	0.59	1 ± 0.46	1.21 ± 1.63	0.30
CTSG	-	-	-	1 ± 1.15	0.36 ± 1.59	0.30	-	-	-	1 ± 0.01	10.3 ± 0.65	0.67
CTSK	1 ± 2.16	2.55 ± 1.40	0.19	1 ± 0.92	1.20 ± 1.02	0.59	1 ± 0.63	1.60 ± 1.51	0.15	1 ± 0.46	1.29 ± 1.63	0.15
CTSV	-	-	-	1 ± 0.01	0.32 ± 9.92	-	-	-	-	-	-	-
CXCL1	1 ± 2.79	9.44 ± 2.38	0.03	1 ± 2.41	1.45 ± 2.42	0.67	1 ± 2.02	1.25 ± 2.53	0.77	1 ± 0.64	3.11 ± 1.82	0.00
CXCL2	1 ± 1.54	2.88 ± 3.26	0.14	1 ± 3.80	2.44 ± 3.50	0.51	1 ± 2.23	0.26 ± 4.26	0.19	1 ± 1.12	0.83 ± 3.74	0.84
CXCL8												
EGF	1 ± 2.23	1.31 ± 1.25	0.72	1 ± 0.57	0.97 ± 1.69	0.94	1 ± 1.05	0.54 ± 1.50	0.21	1 ± 1.83	0.86 ± 1.19	0.74
EGFR	1 ± 1.14	1.10 ± 0.94	0.79	1 ± 0.48	1.04 ± 1.83	0.89	1 ± 0.82	1.22 ± 1.82	0.61	1 ± 0.10	0.75 ± 1.30	0.21
ELANE	1 ± 1.41	3.47 ± 2.13	0.03	1 ± 1.75	2.24 ± 2.55	0.26	1 ± 1.38	2.35 ± 2.30	0.16	1 ± 0.73	3.78 ± 2.07	0.00
F13A1	1 ± 1.18	30.8 ± 2.68	0.01	1 ± 0.80	3.49 ± 2.95	0.23	1 ± 1.60	3.24 ± 1.87	0.12	1 ± 1.11	6.64 ± 2.25	0.01
F3	1 ± 3.27	3.26 ± 1.29	0.26	1 ± 0.46	1.69 ± 1.29	0.06	1 ± 0.93	1.54 ± 1.53	0.28	1 ± 0.21	0.76 ± 1.83	0.08
FGA	1 ± 0.57	1.66 ± 1.27	0.07	1 ± 0.26	1.77 ± 1.44	0.17	1 ± 1.23	1.87 ± 1.90	0.23	1 ± 0.70	1.96 ± 1.39	0.00
FGF10	1 ± 1.22	4.05 ± 2.43	0.04	1 ± 1.05	3.39 ± 1.95	0.06	1 ± 0.78	3.61 ± 2.81	0.03	1 ± 1.14	1.50 ± 2.24	0.00
FGF2	1 ± 1.32	3.50 ± 1.85	0.02	1 ± 1.30	4.25 ± 2.87	0.06	1 ± 1.07	3.23 ± 2.37	0.03	1 ± 0.73	2.73 ± 2.08	0.00
FGF7	1 ± 2.63	0.21 ± 2.11	0.10	1 ± 1.49	0.52 ± 2.18	0.30	1 ± 1.80	0.62 ± 1.93	0.54	1 ± 1.09	0.63 ± 1.60	0.02
FIGF	1 ± 0.93	5.04 ± 2.20	0.02	1 ± 1.41	2.66 ± 2.66	0.25	1 ± 1.50	4.59 ± 2.73	0.03	1 ± 0.21	2.82 ± 1.53	0.00
IL1A	1 ± 2.26	6.56 ± 2.12	0.03	1 ± 1.27	1.54 ± 2.45	0.46	1 ± 0.43	1.05 ± 1.55	0.70	1 ± 0.41	0.83 ± 1.77	0.13
IL1B	1 ± 1.66	4.24 ± 2.67	0.04	1 ± 2.28	1.49 ± 2.40	0.63	1 ± 1.38	1.11 ± 2.78	0.87	1 ± 0.43	2.97 ± 2.17	0.06
IL4	1 ± 1.76	27.7 ± 4.70	0.62	1 ± 1.07	1.23 ± 2.44	0.81	1 ± 0.01	1.37 ± 2.98	-	-	-	-
IL6	1 ± 1.37	3.06 ± 3.15	0.10	1 ± 2.25	1.27 ± 2.39	0.76	1 ± 1.49	0.85 ± 2.68	0.80	1 ± 0.55	3.16 ± 3.61	0.16
IL6ST	1 ± 1.01	1.36 ± 3.80	0.25	1 ± 0.66	0.84 ± 1.64	0.63	1 ± 0.48	0.79 ± 1.57	0.72	1 ± 0.29	0.95 ± 3.58	0.01
ITGA1	1 ± 0.87	2.04 ± 3.84	0.92	1 ± 0.28	0.86 ± 0.92	0.43	1 ± 0.34	0.92 ± 1.51	0.79	1 ± 0.58	1.26 ± 4.35	0.94
ITGA5	1 ± 1.11	1.90 ± 1.34	0.12	1 ± 0.76	1.69 ± 1.57	0.15	1 ± 0.68	2.39 ± 1.20	0.00	1 ± 0.37	1.68 ± 1.37	0.00
ITGB1	1 ± 4.83	3.16 ± 3.33	0.46	1 ± 3.15	1.08 ± 3.16	0.94	1 ± 4.06	0.97 ± 3.44	0.98	1 ± 1.38	0.09 ± 3.05	0.71
ITGB3	1 ± 1.08	2.25 ± 1.34	0.05	1 ± 0.67	1.33 ± 1.39	0.37	1 ± 0.36	1.30 ± 1.41	0.34	1 ± 0.54	1.48 ± 1.44	0.02
ITGB5	1 ± 1.07	1.76 ± 0.92	0.17	1 ± 4.58	23.6 ± 1.64	0.02	1 ± 0.66	1.27 ± 1.34	0.44	1 ± 1.29	1.14 ± 1.85	0.02
ITGB6	1 ± 2.77	0.91 ± 1.57	0.92	1 ± 0.62	0.26 ± 2.28	0.20	1 ± 1.00	0.31 ± 1.97	0.02	1 ± 1.31	0.28 ± 1.91	0.00
MAPK1	1 ± 0.79	2.83 ± 1.17	0.00	1 ± 0.47	3.58 ± 2.08	0.00	1 ± 0.60	3.53 ± 1.98	0.00	1 ± 0.44	3.12 ± 1.91	1.92
MAPK3	1 ± 0.74	1.58 ± 1.40	0.19	1 ± 0.35	1.40 ± 1.19	0.15	1 ± 0.58	1.65 ± 1.82	0.17	1 ± 0.39	1.69 ± 1.26	0.00
MMP1	1 ± 0.95	2.08 ± 1.38	0.08	1 ± 0.48	1.71 ± 2.91	0.92	1 ± 0.14	1.46 ± 2.47	0.64	1 ± 2.24	2.94 ± 1.20	0.05
MMP2	1 ± 1.88	24.5 ± 2.55	0.00	1 ± 0.54	11.1 ± 2.40	0.00	1 ± 0.79	10.4 ± 2.71	0.00	1 ± 0.89	7.72 ± 3.21	3.06
MMP7	1 ± 0.76	2.64 ± 1.53	0.00	1 ± 1.70	0.97 ± 1.82	0.97	1 ± 1.40	1.16 ± 2.55	0.80	1 ± 0.73	1.73 ± 1.67	0.06
MMP9	1 ± 1.07	2.95 ± 1.40	0.01	1 ± 1.42	2.28 ± 2.95	0.23	1 ± 1.71	1.07 ± 2.89	0.92	1 ± 0.94	3.34 ± 2.67	0.01
NGF	1 ± 1.34	1.44 ± 1.34	0.42	1 ± 1.31	1.53 ± 1.85	0.42	1 ± 1.14	1.33 ± 2.85	0.62	1 ± 1.03	2.33 ± 1.36	0.03
PDGFA	1 ± 0.34	2.84 ± 1.37	0.00	1 ± 0.94	3.34 ± 2.46	0.03	1 ± 1.48	4.36 ± 1.96	0.02	1 ± 0.91	2.73 ± 1.33	1.86
PDGFB	1 ± 0.80	0.71 ± 1.10	0.26	1 ± 0.51	0.58 ± 2.10	0.18	1 ± 0.74	0.98 ± 1.54	0.96	1 ± 0.41	0.79 ± 1.32	0.01
PDGFC	1 ± 0.55	1.95 ± 1.40	0.02	1 ± 0.61	1.94 ± 2.23	0.12	1 ± 0.63	3.30 ± 1.81	0.00	1 ± 0.24	2.06 ± 1.72	9.33
PDGFD	1 ± 1.09	1.78 ± 1.88	0.20	1 ± 0.59	0.82 ± 1.76	0.58	1 ± 0.88	0.89 ± 1.56	0.78	1 ± 0.33	0.48 ± 2.74	0.80
PDGFRA	-	-	-	1 ± 6.35	0.01 ± 1.04	0.8	-	-	-	-	-	-
PDFGRB	1 ± 1.83	1.90 ± 1.31	0.39	1 ± 1.21	2.67 ± 1.12	0.08	1 ± 3.15	0.83 ± 1.41	0.86	1 ± 2.05	3.11 ± 2.67	0.09
PLAT	1 ± 0.58	4.57 ± 2.07	0.01	1 ± 1.41	2.01 ± 2.24	0.24	1 ± 1.02	1.67 ± 2.02	0.28	1 ± 0.37	2.52 ± 1.70	0.00
PLAU	1 ± 1.16	2.33 ± 1.34	0.06	1 ± 1.35	1.87 ± 1.91	0.26	1 ± 0.56	0.97 ± 2.34	0.95	1 ± 0.82	1.60 ± 1.63	0.01
PLAUR	1 ± 0.46	1.65 ± 1.20	0.04	1 ± 0.82	1.26 ± 0.89	0.45	1 ± 0.55	1.28 ± 2.16	0.54	1 ± 0.33	1.89 ± 1.53	0.00
PLG	1 ± 0.24	0.46 ± 1.50	0.58	1 ± 0.90	2.92 ± 1.27	0.14	1 ± 1.91	1.27 ± 1.18	0.82	1 ± 2.10	1.01 ± 1.34	0.98
PRTN3	-	-	-	1 ± 1.58	0.86 ± 1.09	0.85	-	-	-	1 ± 0.73	1.44 ± 0.97	0.43
PTEN	1 ± 0.40	2.02 ± 1.16	0.00	1 ± 0.32	1.05 ± 1.31	0.84	1 ± 0.57	1.65 ± 1.74	0.15	1 ± 0.24	1.54 ± 1.36	0.00
PTGS2	1 ± 0.95	4.69 ± 2.58	0.10	1 ± 2.41	1.88 ± 2.99	0.50	1 ± 1.56	0.94 ± 2.05	0.93	1 ± 1.00	1.41 ± 1.87	0.21
RAC1	1 ± 0.83	1.45 ± 1.37	0.27	1 ± 0.37	0.80 ± 0.76	0.23	1 ± 0.53	1.31 ± 1.72	0.42	1 ± 0.23	0.61 ± 2.89	0.21
RHOA	1 ± 2.25	1.49 ± 1.45	0.57	1 ± 0.98	0.80 ± 3.40	0.76	1 ± 0.82	2.60 ± 3.88	0.23	1 ± 0.78	0.87 ± 1.81	0.91
SERPINE1	1 ± 0.64	3.38 ± 1.38	0.00	1 ± 1.65	1.83 ± 1.88	0.33	1 ± 0.54	2.41 ± 1.85	0.02	1 ± 0.44	1.79 ± 1.63	0.00
STAT3	1 ± 4.31	3.54 ± 1.92	0.41	1 ± 3.58	1.10 ± 2.67	0.93	1 ± 2.49	0.88 ± 1.54	0.89	1 ± 2.47	0.63 ± 2.01	0.92
TAGLN	1 ± 4.85	4.99 ± 1.14	0.45	1 ± 0.48	1.08 ± 0.90	0.70	1 ± 0.72	1.78 ± 0.88	0.05	1 ± 0.32	2.17 ± 1.13	0.00
TGFA	1 ± 1.24	2.31 ± 1.15	0.06	1 ± 0.54	1.52 ± 1.55	0.19	1 ± 0.63	1.59 ± 1.34	0.13	1 ± 0.41	1.69 ± 1.70	0.00
TGFB1	1 ± 0.44	1.78 ± 1.60	0.06	1 ± 0.45	2.55 ± 1.44	0.00	1 ± 0.77	2.72 ± 1.57	0.01	1 ± 0.35	2.46 ± 1.53	0.0
TGFB2	1 ± 0.76	3.15 ± 1.35	0.00	1 ± 0.71	1.83 ± 1.56	0.09	1 ± 0.79	1.98 ± 2.18	0.13	1 ± 0.49	2.27 ± 1.57	0.00
TGFBR3	1 ± 0.94	1.11 ± 1.22	0.76	1 ± 0.51	1.78 ± 1.54	0.07	1 ± 0.69	1.90 ± 1.62	0.07	1 ± 0.32	1.66 ± 1.58	0.02
TIMP1	1 ± 2.67	2.54 ± 1.39	0.28	1 ± 0.36	0.71 ± 0.94	0.11	1 ± 0.64	1.58 ± 2.89	0.40	1 ± 0.22	0.68 ± 2.28	0.83
TNF	1 ± 0.91	5.11 ± 2.12	0.01	1 ± 1.01	1.84 ± 2.19	0.23	1 ± 0.97	1.90 ± 1.92	0.16	1 ± 0.94	2.39 ± 2.09	0.00
VEGFA	-	-	-	-	-	-	1 ± 0.67	0.77 ± 2.74	0.68	1 ± 0.01	2.41 ± 1.04	0.03
VEGFB	1 ± 4.65	10.3 ± 1.30	0.25	1 ± 0.73	0.87 ± 1.82	0.72	1 ± 0.96	1.04 ± 2.03	0.93	1 ± 0.91	0.89 ± 1.69	0.15
VEGFC	1 ± 0.66	1.18 ± 1.42	0.58	1 ± 0.55	1.19 ± 1.03	0.47	1 ± 0.56	1.78 ± 1.72	0.10	1 ± 0.23	1.59 ± 1.28	0.07
WISP1	1 ± 0.68	1.60 ± 1.39	0.13	1 ± 0.63	1.55 ± 1.11	0.12	1 ± 0.42	1.60 ± 1.77	0.53	1 ± 0.50	1.62 ± 1.50	0.02
WNT5A	1 ± 0.78	3.66 ± 1.35	0.00	1 ± 0.34	1.02 ± 1.99	0.94	1 ± 0.38	1.55 ± 1.99	0.07	1 ± 0.51	1.15 ± 2.10	0.00

## Data Availability

The data that support the findings of this study are available from the corresponding author, W.P., upon reasonable request.

## Supplementary Materials

The following are available online at https://www.mdpi.com/article/10.3390/ijms22168659/s1.
