# Supplementary material for: Single Dose of N-Acetylcysteine in Local Anesthesia Increases Expression of HIF1α, MAPK1, TGFβ1 and Growth Factors in Rat Wound Healing
Source: Int J Mol Sci. 2021 Aug 12;22(16):8659. doi: 10.3390/ijms22168659 (PMC8395485; doi:10.3390/ijms22168659)
Supplement: Supplementary file 1 [file ijms-22-08659-s001.zip › Supplement File 1.pdf]

Supplement File 1 – QuPath script for automated assessment of immunohistochemical staining with anti-MPO, anti-CD31 and anti-CD68 antibodies

Script for anti-MPO

```
setImageType('BRIGHTFIELD_H_DAB');

setColorDeconvolutionStains('{"Name" : "H-DAB default", "Stain 1" : "Hematoxylin", "Values 1" : "0.65111 0.70119 0.29049 ", "Stain 2" : "DAB", "Values 2" : "0.26917 0.56824 0.77759 ", "Background" : " 255 255 255 "}');

selectAnnotations();

runPlugin('qupath.imagej.detect.nuclei.PositiveCellDetection', '{"detectionImageBrightfield": "Hematoxylin OD", "requestedPixelSizeMicrons": 0.5, "backgroundRadiusMicrons": 8.0, "medianRadiusMicrons": 0.0, "sigmaMicrons": 1.5, "minAreaMicrons": 10.0, "maxAreaMicrons": 400.0, "threshold": 0.1, "maxBackground": 2.0, "watershedPostProcess": true, "excludeDAB": true, "cellExpansionMicrons": 5.0, "includeNuclei": true, "smoothBoundaries": true, "makeMeasurements": true, "thresholdCompartment": "Cytoplasm: DAB OD mean", "thresholdPositive1": 0.2, "thresholdPositive2": 0.4, "thresholdPositive3": 0.6, "singleThreshold": true}');
```

Script for anti-CD31 and anti-CD68

```
setImageType('BRIGHTFIELD_H_DAB');

setColorDeconvolutionStains('{"Name" : "H-DAB default", "Stain 1" : "Hematoxylin", "Values 1" : "0.65111 0.70119 0.29049 ", "Stain 2" : "DAB", "Values 2" : "0.26917 0.56824 0.77759 ", "Background" : " 255 255 255 "}');

selectAnnotations();

runPlugin('qupath.imagej.detect.nuclei.PositiveCellDetection', '{"detectionImageBrightfield": "Hematoxylin OD", "requestedPixelSizeMicrons": 0.5, "backgroundRadiusMicrons": 8.0, "medianRadiusMicrons": 0.0, "sigmaMicrons": 1.5, "minAreaMicrons": 10.0, "maxAreaMicrons": 400.0, "threshold": 0.1, "maxBackground": 2.0, "watershedPostProcess": true, "excludeDAB": true, "cellExpansionMicrons": 5.0, "includeNuclei": true, "smoothBoundaries": true, "makeMeasurements": true, "thresholdCompartment": "Cell: DAB OD mean", "thresholdPositive1": 0.2, "thresholdPositive2": 0.4, "thresholdPositive3": 0.6, "singleThreshold": true}');
```
