# Supplementary material for: Single Dose of N-Acetylcysteine in Local Anesthesia Increases Expression of HIF1α, MAPK1, TGFβ1 and Growth Factors in Rat Wound Healing
Source: Int J Mol Sci. 2021 Aug 12;22(16):8659. doi: 10.3390/ijms22168659 (PMC8395485; doi:10.3390/ijms22168659)
Supplement: Supplementary file 1 [file ijms-22-08659-s001.zip › Supplement Table 1.pdf]

Supplement Table 1 – Gene symbols, full names and primers sequences

Symbol	Gene name	Forward Primer sequence	Reverse Primer seguence
ACTB	Beta-actin	GGGAAATCGTGCGTGACATT	GCGGCAGTGGCCATCTC
GAPDH	Glyceraldehyde 3-phosphate dehydrogenase	TGATTCTACCCACGGCAAGTT	TGATGGGTTTCCCATTGATGA
LDHA	Lactate dehydrogenase A	CCGTTACCTGATGGGAGAAA	ACGTTCACACCACTCCACAC
NONO	Non-POU Domain Containing Octamer Binding	AAATGGCTATGGGAGGTGCC	CAGCTTGGCCAAAACGTTCA
PPIH	Peptidyl-prolyl isomerase H	GCCAGTATTTACCGGGGTCC	ACATCCTTTCACCTGGTTCAGT
GDC	Solute carrier family 25 (mitochondrial carrier; Graves disease autoantigen), member 16	AAGAGCCATCTTGGGCTGAC	TGAGGGGGTACAACATGTGC
ACTA2	Actin alpha 2 skeletal muscle	CATCCGACCTTGCTAACGGA	GTCCAGAGCGACATAGCACA
ACTC1	Actin, alpha 1 skeletal muscle	ATTATTGCTCCCCCTGAGCG	CTGAGTGTAAGGTAGCCGCC
ANGPT1	Angiopoietin 1	ACGCTGAACGGTTACACAGA	TTCCCGTCGTGTTCTGGAAG
CCL2	C-C Motif Chemokine Ligand 2	ATGCAGTTAATGCCCCACTC	TTCCTTATTGGGGTCAGCAC
CD40LG	TNF-Related Activation Protein	GGCAGCGAATACCCACAGTT	CACTTGGCTTGCTTCAGTCAC
CDC42	Cell division control protein 42 homolog	CGGAGAAGCTGAGGTCAACA	GCGTTCATAGCAGCACACAC
CDH1	E-cadherin	CGTGGATGTGGTAGACGTGAA	TTCTCCGCAGGCACAAAAAT
COL14A1	Collagen Type XIV Alpha 1 Chain	GAATTGGGGGCCCAAACAAC	CGGGTTAGACACGAAGGAGG
COL1A2	Collagen Type I Alpha 2 Chain	AGGTGAAGCTGGCAACATCG	GGATGGCCTTTCTCACCAGGTT
COL3A1	Collagen Type III Alpha 1 Chain	CACCCCTCTCTTATTTTGGCAC	AGACTCATAGGACTGACCAAGGTAGTT
COL4A1	Collagen Type IV Alpha 1 Chain	TGGAACGAAAGGGACACGAG	TCACCGGGATCTCCCTTCAT
COL4A3	Collagen Type IV Alpha 3 Chain	CCCAAAGGCATCAGGGGAAT	GTGCATCATAACATTTTACTGGACC
COL5A1	Collagen Type V Alpha 1 Chain	GTCTGAGGGAGCCAGAATCAC	GCCTTATCGTAGCTGCCTGT
COL5A3	Collagen Type V Alpha 3 Chain	CTCCAGCTGTCCAGAGTGACTG	CATGTGGGGACGCTGAGAAG
CSF2	Granulocyte-Macrophage Colony-Stimulating Factor	AAATGACATGCGTGCTCTGG	TGGCTATCATGGTCAAGGCG
CSF3	Granulocyte Colony-Stimulating Factor	CTGTGGAGCAGGGAAGGAGA	GCGGCAGGAATCAATACTCG
CSK	Tyrosine-protein kinase	AGCTGAGTCCTACCTTCCCA	AAGGCCGGTCTGTTACTTGG
CTGF	Connective tissue growth factor	CTGTGGGAGAAAACACCCCA	CACTCTTCCAGGAGGCTCAC
CTNNB1	Catenin Beta 1	CGTGAGCCTTAAGCTCCCATT	GTATAGACAGACGCCCCTGC
CTSG	Cathepsin G	AAGCAGCAGTGGTAACCCTC	TGAACTTGGATGGATGCCCA
CTSK	Cathepsin K	TCCTCAACAGTGCAAGCGAA	CCAGCGTCTATCAGCACAGA
CTSV	Cathepsin V	GTGTTCCGTGAGCCTCTGTTTC	CGGAACATCTGTCCTTCAAGAGC

CXCL1	C-X-C Motif Chemokine Ligand 1	CAATGAGCTGCGCTGTCAGT	TTGAAGTGAATCCCTGCCACT
CXCL2	C-X-C Motif Chemokine Ligand 2	GCGCCCAGACAGAAGTCATA	CAGGTACGATCCAGGCTTCC
CXCL8	C-X-C Motif Chemokine Ligand 8 (Interleukin 8)	GAAGATAGATTGCACCGA	CATAGCCTCTCACACATTTC
EGF	Epidermal growth factor	AACACGGAGGGAGGCTACAA	CCCTGGGGGAGCTTAGAGAAAG
EGFR	Epidermal growth factor receptor	CCACCAAGACAGGCGACG	AGCAGTAGCTTGGTTCTCGC
ELANE	Neutrophil Elastase	TGGTGGACCCTTGGTCTGTA	TAAGTGGCCGGTCATCATGG
F13A1	Coagulation Factor XIII A Chain	GCAGAGGGAACCCCATCAAA	GGGATGCCGTAGGCATAGAC
F3	Tissue Factor, Coagulation Factor III	TGCTTCTTCGTACAAGCCGT	GAGGTCTCGGTAACAGTGCC
FGA	Fibrinogen Alpha Chain	TGCCTCATCCTGAGCTTGG	CCACGAATATCTCCTCCTGCT
FGF10	Fibroblast Growth Factor 10	CGGAGTTGTTGCCGTCAAAG	GCCACATACATTTGCCTGCC
FGF2	Fibroblast Growth Factor 2	TTCACAGCCTGTGCTCTAGGG	GATCGGGTCAGGTTTTGGAAA
FGF7	Fibroblast Growth Factor 7	AGGGAGGGGTGGCTTTTTAG	CGTGCATTTGCTGCTTGACT
FIGF	Vascular Endothelial Growth Factor D	TCTGGGTGTCATGTTACAGGC	CAGGCTGGAACCCAACTATGG
HBEGF	Heparin Binding EGF Like Growth Factor	AGGACTTGGAAGGGACCGAT	GGAACCGCCATCTCAGAAGT
HGF	Hepatocyte Growth Factor	ACAGCTTTTTGCCTTCGAGC	GCAAGAATTTGTGCCGGTGT
HIF1A	Hypoxia Inducible Factor 1 Subunit Alpha	ATCTGAGGACACGAGCTGCC	GCATCGGGCTCTTTCTTAAGC
IFNG	Interferon Gamma	ATTCATGAGCATCGCCAAGTTC	TGACAGCTGGTGAATCACTCTGAT
IGF1	Insulin Like Growth Factor 1	AATGTGCGGTTCTGTGGGAG	CTCATCCACAATGCCCGTCT
IL10	Interleukin 10	TTGAACCACCCGGCATCTAC	CCAAGGAGTTGCTCCCGTTA
IL1A	Interleukin 1 alpha	CCATCCAACCCAGATCAGCA	TCTCCTCCCGATGAGTAGGC
IL1B	Interleukin 1 beta	GACCTGTTCTTTGAGGCTGACA	CTCATCTGGACAGCCCAAGTC
IL4	Interleukin 4	GTACCAGACGTCCTTACGGC	CAGACCGCTGACACCTCTAC
IL6	Interleukin 6	TAGTCCTTCCTACCCCAACTTCC	TTGGTCCTTAGCCACTCCTTC
IL6ST	Interleukin 6 Signal Transducer	CCGACTTCCCTGACGTTGT	CACGGGAACTGAAAGAACGC
ITGA1	Integrin Subunit Alpha 1	CTAAGCAGACACAGGTCGGG	CGATTCTCCGTCGGTCACAA
ITGA5	Integrin Subunit Alpha 5	CCCTCGTTTACACATGCCCT	ATGAATCTTTGCAGGCGGGA
ITGB1	Integrin Subunit Beta 1	ACAAGAGTGCCGTGACAACT	CTTCCGCACGCATCATTGAG
ITGB3	Integrin Subunit Beta 3	TGACCCGCTTCAATGACGAA	ATGGGTCTTGGCATCAGTGG
ITGB5	Integrin Subunit Beta 5	CACGGTCCATCATCTCTCGG	CATGGAGAGGGAGAGGTCCA
ITGB6	Integrin Subunit Beta 6	AGCTCAAGTTACTTTTCAAAGCAGT	TCTGTCGACCTACGCTGAGA
MAPK1	Mitogen-activated protein kinase 1	TGGTTCTACCGGCGGTTAGT	AACGAGGAGGGAGGACAACAC

MAPK3	Mitogen-activated protein kinase 3	CCAGCTCTGGTAGACGGTTC	GCTCCTCAGGGAAACTAGCC
MMP1	Matrix metalloproteinase-1	AGCTCATACAGTTTCCCCGT	GCCTCAGCTTTTCAGCCATC
MMP2	Matrix metalloproteinase-2	TTTGCTCGGGCCTTAAAAGTAT	CCATCAAACGGGTATCCATCTC
MMP7	Matrix metalloproteinase-7	ACAGACTTGCCTCGGTTCTT	GTCTCCGTGATCTCCCCTTG
MMP9	Matrix metalloproteinase-9	GTGCCCTGGAACTCACACAAC	CCAGAAGTATTGTCATGGCAGAA
NGF	Nerve growth factor	ACGCCCCTTCTCCTCTCACAAGTG	GGCTGTGTCAAGGGAATGCTGAAG
PDGFA	Platelet-derived growth factor subunit A	CAGGACAGGACGCGTAGAAC	CGGGTTGCTCGAGGTCTTAG
PDGFB	Platelet-derived growth factor subunit B	AGAGCCTGGCATTTAGCCTG	GGTGCCATGAGAGTTCCTCC
PDGFC	Platelet-derived growth factor subunit C	CCGGCATGAGAGAGTTGTCA	TCAGCTGGATCCGCACATTT
PDGFD	Platelet-derived growth factor subunit D	CAGAGCGCATCCATCAAAGC	TCTTCCTCTGACAACGGTGC
PDGFRA	Platelet-derived growth factor receptor Alpha	GAACCCCTCTGGCCTTCATTC	TTGACCTCTTTCGTGGCCTG
PDFGRB	Platelet-derived growth factor receptor Beta	GTAAAGCCCACAGTGGTGTGA	CCCAGGAGCAATAGCACGAA
PLAT	Plasminogen activator, tissue type	GCATCATCCACACAGAAGGGA	TTCATCTCTGCAGGTCGCTC
PLAU	Plasminogen Activator, Urokinase type	ACTGCTTCGTGAATCAGCCA	TGGAAGGCCAGAGTTTCGTC
PLAUR	Plasminogen Activator, Urokinase receptor	AGAGCTTACCACCGAATGGC	CAATGAGGACGTCTCTTCGTAGG
PLG	Plasminogen	GCCCAACCTACCAATGTCTGA	TTGGTAGTGGACGATGTGCC
PRTN3	Proteinase 3	AATCCGCCCATCCCTCAAAG	TGCAGGGATGCCACATAAGG
PTEN	Phosphatase And Tensin Homolog	ATTCCCAGTCAGAGGCGCTA	TCACCTTTAGCTGGCAGACC
PTGS2	Prostaglandin-Endoperoxide Synthase 2	CTCAGCCATGCAGCAAATCC	GGGTGGGCTTCAGCAGTAAT
RAC1	Rac Family Small GTPase 1	TGCTCAGAAAATGGCGGAGT	GTTCTCAGCACAACGCAACT
RHOA	Ras Homolog Family Member A	GTAGCCTACCACTGCGAAGC	CCGTGGGCACATAAACCTCT
SERPINE1	Plasminogen Activator Inhibitor 1	CACTACGCCTTCACTCAGCA	TCGGGCGATCATTCGATCTG
STAT3	Signal transducer and activator of transcription 3	TGGAAGAGGCGGCAGCAGATAGC	CACGGC CCCCATTCCCACAT
TAGLN	Transgelin	ATCCTATGGCATGAGCCGTG	CAGGCTGTTCACCAACTTGC
TGFA	Transforming growth factor alpha	CGCACGTACACACACCAAAC	TCAGTGCATCATATACAGAAGTCAGAA
TGFB1	Transforming growth factor beta 1	AGGACCTGGGTTGGAAGTGG	AGTTGGCATGGTAGCCCTTG
TGFB2	Transforming growth factor beta 2	GTGTTTTCATCATGCTGGCT	CGTGAAGTGGCTGTTGATCT

TGFBR3	Transforming Growth Factor Beta Receptor 3	GCCTAGCTGAACCAAGGTGT	CTCCCCACAAAGGCTAGCAA
TIMP1	Metallopeptidase inhibitor 1	CACCCCTCGCATGGACATTTA	CCGGAAACCTGTGGCATTTC
TNF	Tumor necrosis factor	GACCCTCACACTCAGATCATCTTCT	TGCTACGACGTGGGCTACG
VEGFA	Vascular endothelial growth factor A	ACTCATCAGCCAGGGAGTCT	GAGCCCAGAAGTTGGACGAA
VEGFB	Vascular endothelial growth factor B	GTGGTCAAACAACTCGTGCC	CCTGTGGAGTCCGAAAAGCA
VEGFC	Vascular endothelial growth factor C	ATGTGGGGAAGGAGTTTGGA	GTTTGGGGCCTTGAGAGAGA
WISP1	WNT1-inducible-signaling pathway protein 1	GCATAGCCTACACTAGCCCC	TTCCCTGCCTTGATGTGTGG
WNT5A	Wnt Family Member 5A	GCTTCCGCCCATGGAAGATA	CCTAACAGTCCGGTTTGGGT
